# Supplementary material for: EGFR-dependent TOR-independent endocycles support Drosophila gut epithelial regeneration
Source: Nat Commun. 2017 May 9;8:15125. doi: 10.1038/ncomms15125 (PMC5436070; doi:10.1038/ncomms15125)
Supplement: Supplementary Information — Supplementary Figures. [file ncomms15125-s1.pdf]

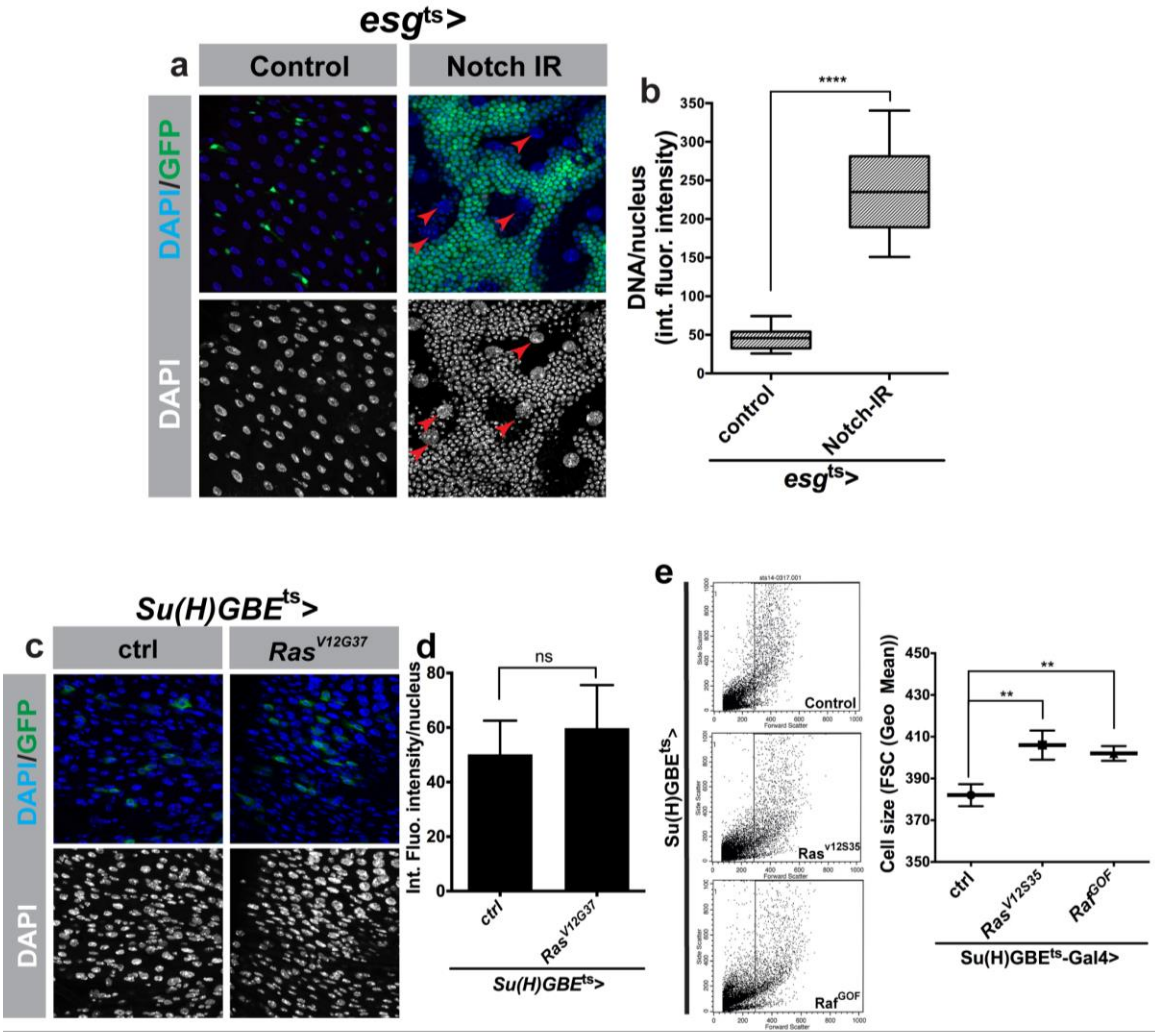

Supplementary Figure 1 | Epithelial loss induces compensatory EC hypertrophy and hyperploidy

(a, b) RNAi-mediated knockdown of *Notch* in *esg*-expressing cells for 5 days depletes the epithelium of ECs by preventing the differentiation of progenitor cells. Guts were stained with DAPI to assess EC ploidy. GFP driven by *esg-Gal4*^{ts} marks diploid progenitor cells. (b) DNA content/nucleus was quantified by measuring integrated DAPI fluorescent intensity. 80 nuclei from 10 midguts/genotype were quantified. Blocking EC differentiation with Notch RNAi (IR) gradually depleted the epithelium of ECs, and remaining ECs developed huge hyperpolyploid nuclei.
(c, d) Overexpression of *Ras*^{V12G37} in EBs and pre-ECs for 3 days driven by *Su(H)GBE-Gal4*. This activated allele of *Ras* cannot signal to *Raf*, but can activate *Pi3K*. Nuclei are indicated by DAPI staining (blue), GFP marks expressing EBs and pre-ECs (green). (d) Quantification of integrated fluorescence intensity/nucleus for c. 100 nuclei from 10 midguts/genotype were quantified. *Ras*^{V12G37} had no significant effect on endoreplication.
(e) Midguts in which *Ras*^{V12S35} overexpressing, *Raf*^{GOF} overexpressing, or control

GFP marked cells driven by *Su(H)GBE-Gal4* were digested into single cell suspensions. Cell volumes for the GFP+ EBs and pre-ECs in each sample were measured using the forward scatter parameter of FACS. Error bars represent standard deviations. The Student's T-test was used to determine statistical significance. (****: $p<0.0001$).

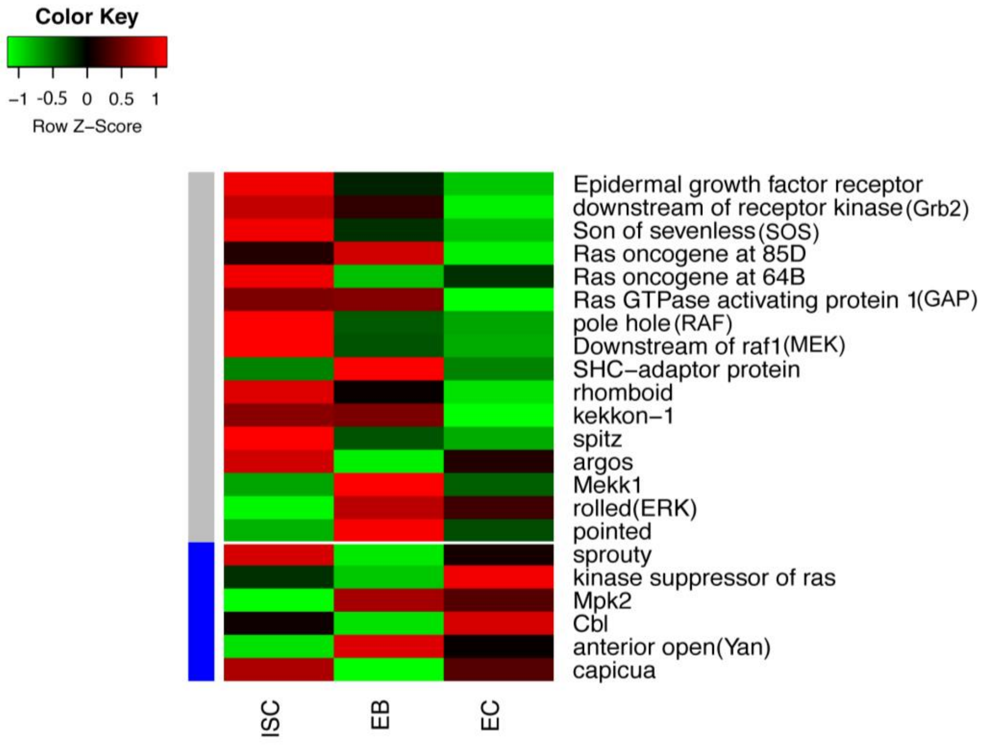

Supplementary Figure 2 | Transcript levels of MAP kinase components in different cell types of the adult midgut

100 midguts/sample were digested by Collagenase and Trypsin into single cell suspensions. Then GFP-marked ISCs, EBs or ECs were purified by FACS using GFP expressed by the *Dl-Gal4, Su(H)GBE-Gal4,* or *MyoIA-Gal4* drivers, respectively. mRNA was purified from each cell type and sequenced by Hiseq 2000. The absolute transcription value was normalized to RPKM. See (40) for protocol and further data. The heatmap illustrates selected genes in the EGFR/Map kinase pathway. Colors indicate mRNA gene expression levels (red: high, green: low). (Raw data from FlyGutSeq). (Z-score scale is indicated in the up-left corner of figure).

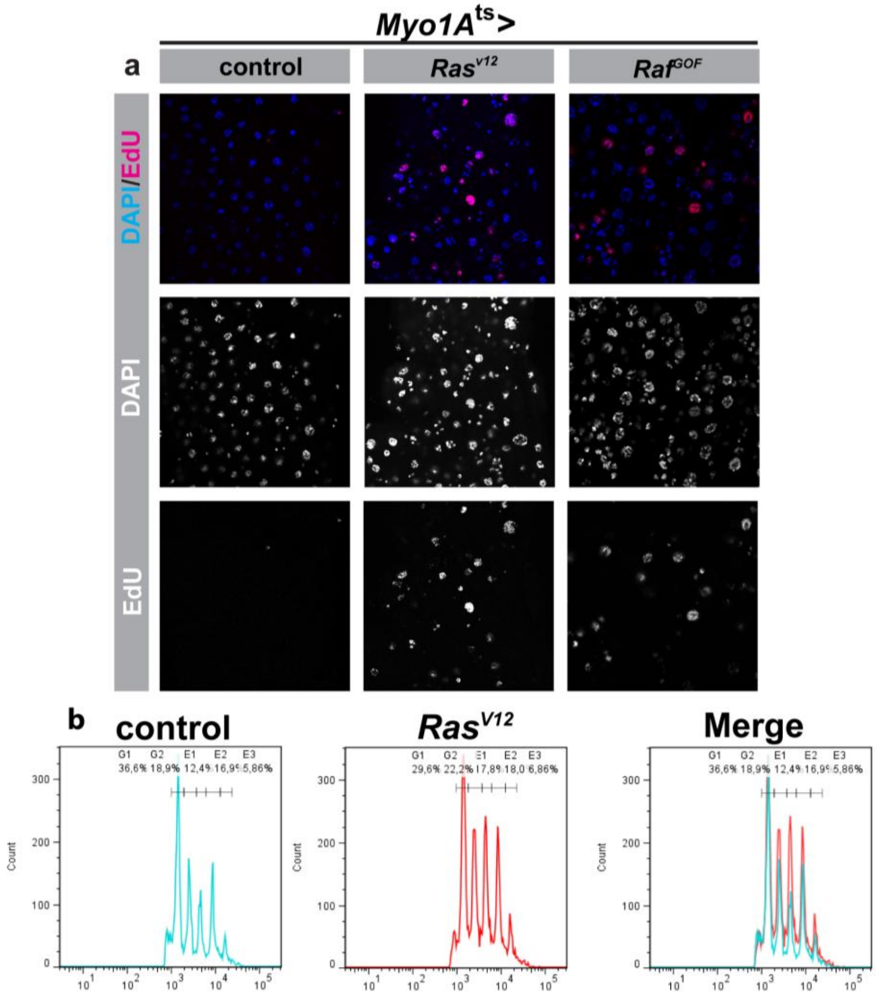

Supplementary Figure 3 | Ras/Raf activity in ECs promotes endocycle progression

Overexpression of *Ras^{V12}* or *Raf^{GOF}* by *Myo1A-Gal4^{ts}* increased DNA re-replication in ECs. Flies were shifted to 29°C for 1 day to induce the indicated transgenes. After dissection, midguts were incubated with EdU *in vitro* for 2h, and then fixed. Midguts were stained with DAPI (blue) and incorporated EdU was fluorescently labeled (red). (a) Wild-type control guts displayed very little EdU incorporation. Overexpression of *Ras^{V12}* or *Raf^{GOF}* strongly induced endoreplication, as indicated by extensive EdU incorporation. (b) Analysis of EC ploidy by FACS. Midguts were dissected from *Ras^{V12}* overexpressing and control flies prior to digestion with collagenase and trypsin to generate a single cell suspension. Nuclei were purified from the cells before staining with DAPI, a specific DNA-binding dye, and then analyzed by FACS. Compared to controls, *Ras^{V12}* overexpression resulted in more nuclei with higher DNA copy number. Left: control (blue), middle: overexpression of *Ras^{V12}* (red) by *Myo1A-Gal4*, right: Merge. All experiments were repeated three times.

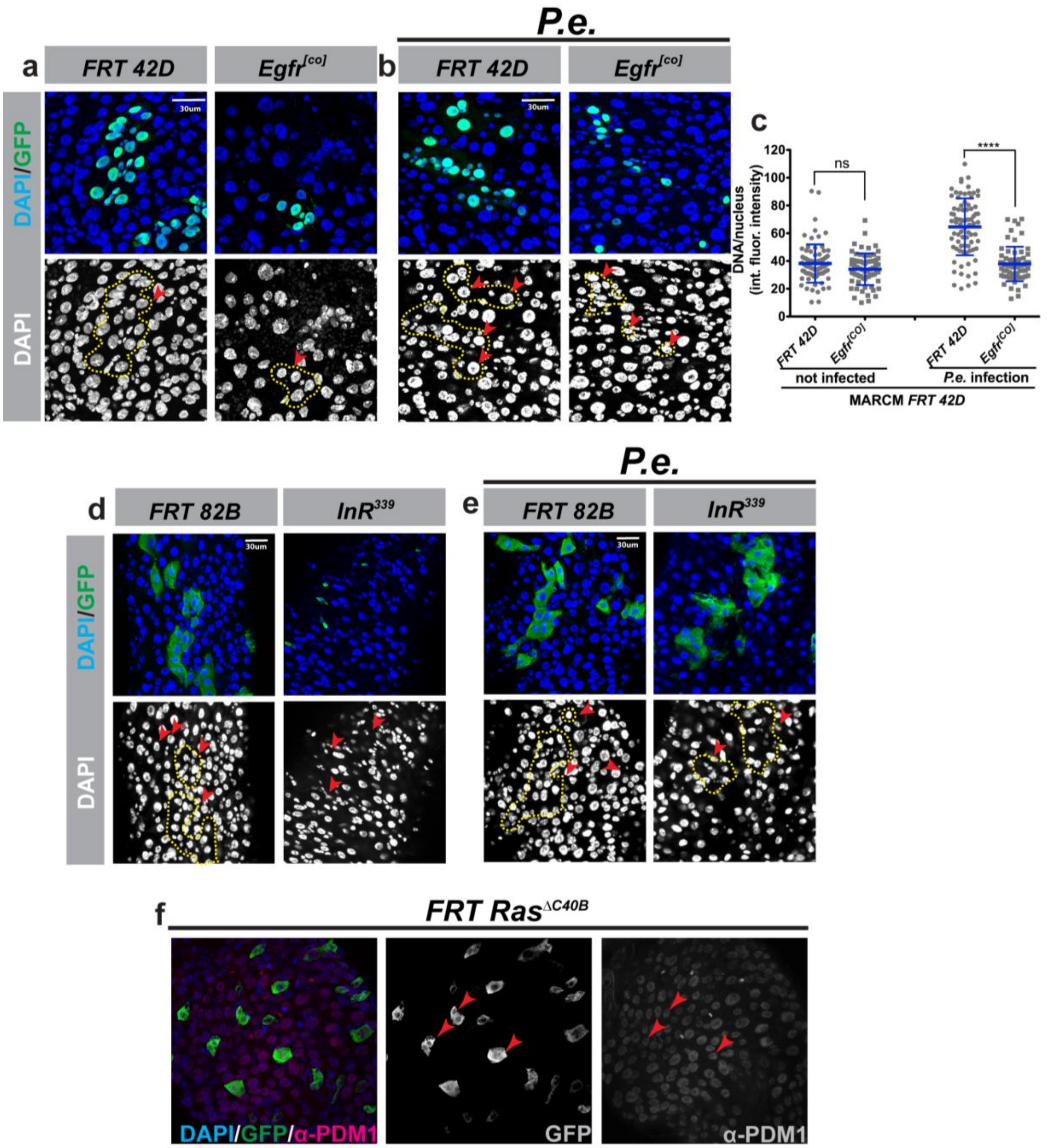

Supplementary Figure 4 | *EGFR, InR* and *Ras* function assayed in midgut MARCM clones

EGFR^{co}, *InR*^{339}, or *Ras85D*$^{\Delta C40B}$ mutant clones were made using the MARCM system. Several days after clone induction, midguts were dissected. DNA was stained with DAPI (blue). Clones were marked with GFP (green), and are outlined by yellow dashed lines. All experiments were repeated three times.

(a) FRT control and *EGFR*^{co} mutant clones 3 days after induction. (b) 2 days after clone induction flies were infected by *P.e.* for 24h. (c) Quantification of DNA/nucleus for GFP+ clone cells from a-b. 80 GFP+ nuclei from 10 midguts were scored for each genotype. Error bars represent standard deviations and the Student's T-test was used to determine statistical significance. (****: $p<0.0001$, ns: $p>0.05$). Loss of *EGFR* resulted in lower ploidy only after *P.e.* infection.

(d) FRT control and *InR*^{339} mutant clones. (e) 2 days after clone induction, FRT control and *InR* mutant flies were infected by *P.e.* for 24h. *InR*$^{-/-}$ mutant clones were arrested as one or two diploid cells under normal conditions, but grew into large clones with polyploid ECs after *P.e.* infection.

(f) 5 day old *Ras85D*$^{\Delta C40B}$ null mutant clones generated using the MARCM system. DAPI stain shows DNA content (blue), and clones were marked with GFP (green). *PDM1* antibody staining indicates differentiated ECs (red). Many *Ras*$^{-/-}$ mutant cells are *PDM1* positive.

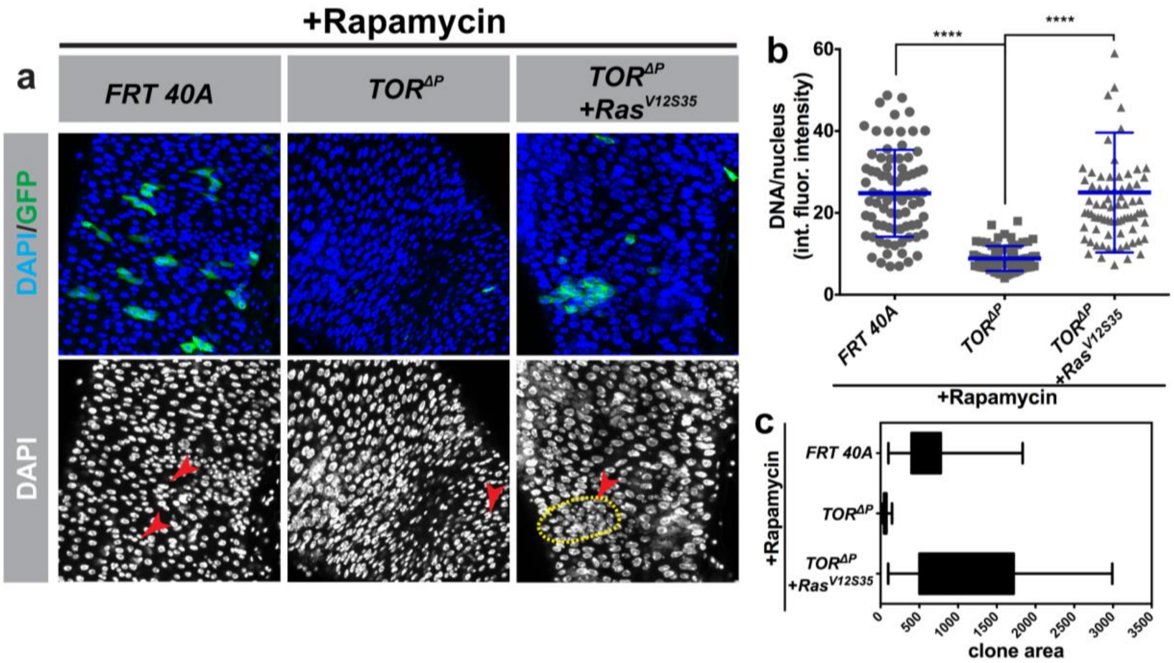

Supplementary Figure 5 | *Ras* activity bypasses *TOR* to induce cell division, endoreplication and clonal growth

(a) $TOR^{\Delta P}$ mutant clones either expressing or not expressing Ras^{V12S35} were generated using the MARCM system. All flies were fed Rapamycin for two days prior to clone induction to ensure strong suppression of *TOR* activity. 5 days after clone induction, with continuous exposure to Rapamycin, midguts were harvested and labeled with DAPI (blue). Clones are marked with GFP (green). Clonal boundaries are outlined with yellow dashed lines. (b) Quantification of DNA content of clone cells by measuring the integrated DAPI intensity per nuclues for a. 80 GFP+ nuclei from clone in 10 midguts per genotype were counted. (c) Quantification of clone areas. Error bars represent standard deviations and the Student's T-test was used to determine statistical significance. (****: $p<0.0001$).Ras^{V12S35} overexpression enabled midgut cells to divide, grow, and endoreplicate in the absence of *TOR* activity. This experiment was repeated three times.

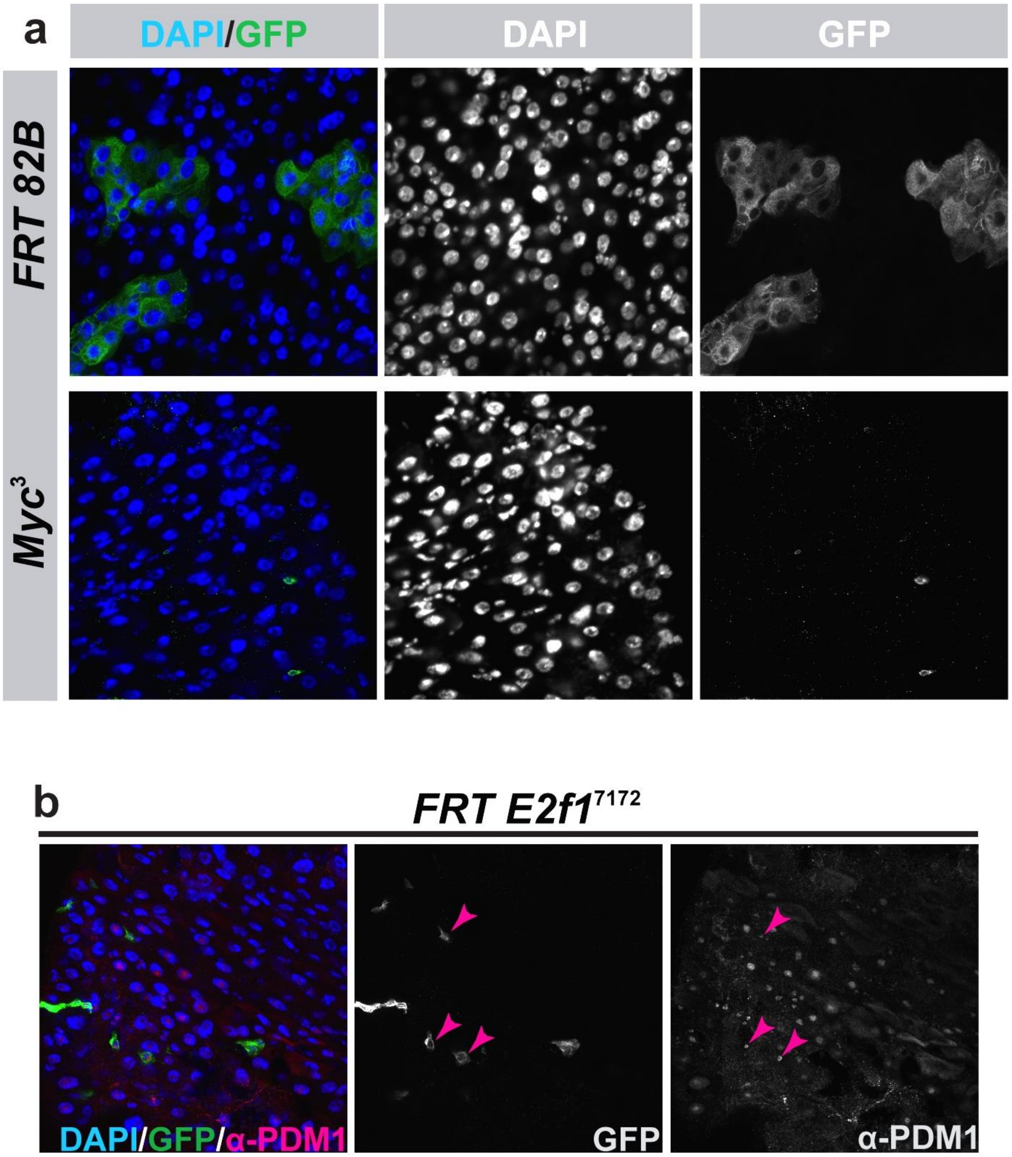

Supplementary Figure 6 | Deletion either *E2f1* or *Myc* in midgut cell clones blocks both cell growth and endoreplication

Myc^{3} or *E2f1*^{7172} null mutant clones were generated in the midgut using the MARCM technique. 7 days after clone induction, midguts were dissected, fixed and stained for DNA (blue) with DAPI. GFP (green) marks mutant cells. *E2f1* samples were stained with *PDM1* antibody to detect EC differentiation. (a) *FRT* control and *Myc* mutant clones. *Myc*$^{-/-}$ clones arrest as single small diploid cells. (b) *E2f1* mutant clones. Some of *E2f1* clone cells are *PDM1*-positive and therefore are differentiated ECs, despite their very small size. All experiments were repeated three times.

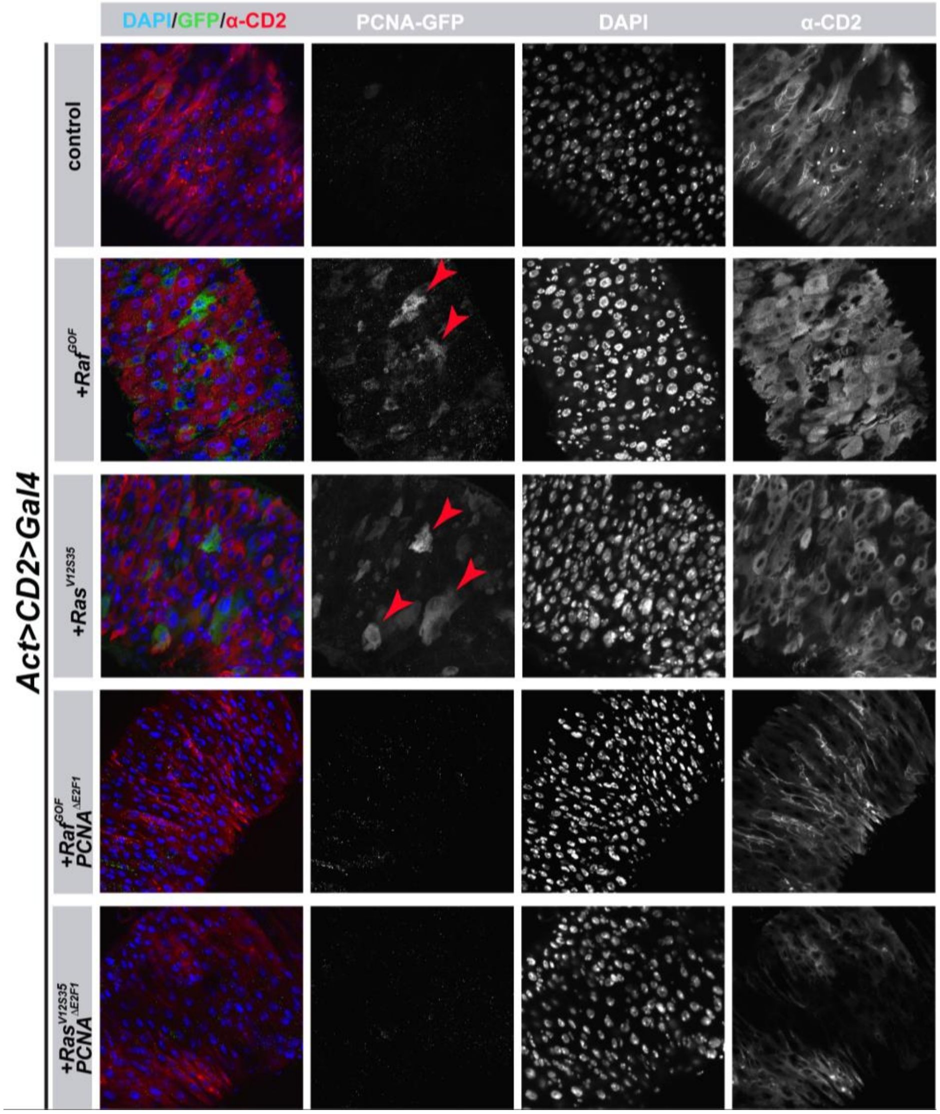

Supplementary Figure 7 | Ras/Raf signaling induces *E2f1* transcriptional activity

The *hs-Flp Act>CD2>Gal4* driver ("flip-out Gal4") was used to force widespread expression of *Ras*^{V12S35} or *Raf*^{GOF} in flies carrying the *PCNA-GFP* reporter, which is *E2f1* responsive, or the *PCNA-GFP*$^{\Delta E2f1}$ reporter, which lacks *E2f1* binding sites in its promoter and is not *E2f1*-responsive. 3 days after clone induction, midguts were dissected, fixed, and analyzed. The midguts were labeled with DAPI to visualize DNA (blue), anti-*CD2* antibody to indicate non-clone cells (red) and anti-GFP (green) to detect *PCNA*-GFP reporter expression. Overexpression of *Ras*^{V12S35} or *Raf*^{GOF} induced *PCNA-GFP* reporter transcription, indicating stimulation of *E2f1* transcriptional activity. Consistent with this conclusion, transcription failed to be induced in flies with the *E2f1* insensitive reporter, *PCNA-GFP*$^{\Delta E2f1}$. This experiment was repeated three times.

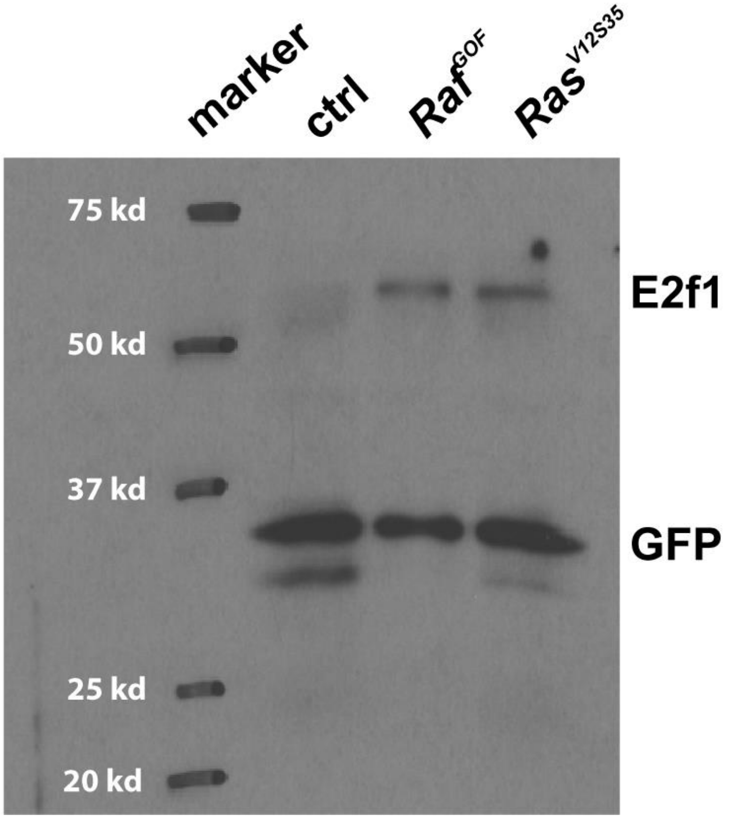

Supplementary Figure 8 | *E2f1* protein level under Ras/MAPK signaling activity.

Experimental design and protein extraction method is described in legend to Fig. 6g. This uncropped blot displays levels of *E2f1* and GFP protein.
